# Water–fat magnetic resonance imaging quantifies relative proportions of brown and white adipose tissues: *ex-vivo* experiments

**DOI:** 10.1117/1.JMI.5.2.024007

**Published:** 2018-06-29

**Authors:** Jadranka Stojanovska, Carey N. Lumeng, Cameron Griffin, Diego Hernando, Udo Hoffmann, Jonathan W. Haft, Karen M. Kim, Charles F. Burant, Kanakadurga Singer, Alex Tsodikov, Benjamin D. Long, Matthew A. Romano, Paul C. Tang, Bo Yang, Thomas L. Chenevert

**Affiliations:** a Michigan Medicine, Division of Cardiothoracic Radiology, Department of Radiology, Ann Arbor, Michigan, United States; b Michigan Medicine, Department of Pediatrics and Molecular Physiology, Ann Arbor, Michigan, United States; c Michigan Medicine, Division of Pediatric Endocrinology, Ann Arbor, Michigan, United States; d University of Wisconsin, Wisconsin Institutes for Medical Research, Medical Physics Department, Madison, Wisconsin, United States; e Massachusetts General Hospital, Department of Radiology, Boston, Massachusetts, United States; f Michigan Medicine, Frankel Cardiovascular Center, Department of Cardiac Surgery, Ann Arbor, Michigan, United States; g University of Michigan, Ann Arbor, Michigan, United States; h Michigan Medicine, Division of Pediatric Endocrinology, Department of Pediatrics and Communicable Diseases, Ann Arbor, Michigan, United States; i School of Public Health, Ann Arbor, Michigan, United States; j University of Michigan Medical School, Cardiovascular Center, Ann Arbor, Michigan, United States; k Michigan Medicine, Cardiovascular Center, Ann Arbor, Michigan, United States; l Michigan Medicine, Department of Radiology–MRI, Ann Arbor, Michigan, United States

**Keywords:** multiecho Dixon, brown adipose tissue, white adipose tissue, ex-vivo study, T2*

## Abstract

Quantifying the amount of brown adipose tissue (BAT) within white adipose tissue (WAT) in human depots may serve as a target to combat obesity. We aimed to quantify proton density fat fraction (PDFF) of BAT and WAT in relatively pure and in mixed preparation using water–fat imaging. Three *ex-vivo* experiments were performed at 3 T using excised interscapular BAT and inguinal/subcutaneous WAT from mice. The first two experiments consisted of BAT and WAT in separate tubes, and the third used mixed preparation with graded quantities of BAT and WAT. To investigate the influence of partial volume on PDFF metrics, low ($2.66\;{\mathrm{mm}}^{3}$) and high spatial resolution ($0.55\;{\mathrm{mm}}^{3}$ acquired voxels) in two orthogonal three-dimensional sections were compared. The low-resolution acquisitions are corrected for T2* and multipeak lipid spectrum, thus considered “quantitative,” whereas the high-resolution acquisitions are not corrected but were performed to better spatially segment BAT from WAT zones. As potential BAT metrics, we quantified the average PDFF and the volume of tissue having PDFF ≤50% (${\mathrm{VOL}}_{\mathrm{PDFF}\leq 50\%}$) based on the PDFF histogram. In the first experiment, the average PDFF of BAT was 23±6% and 21±7.6% and the average PDFF of WAT was 76±7% and 87±7% using high- and low-resolution techniques, respectively. A similar trend with excellent reproducibility in average PDFF of BAT and WAT was observed in the second experiment. In the third experiment over the four acquisitions, the BAT-dominant tube demonstrated lower PDFF (mean ± SD) of 55±2% than WAT-dominant (69±4%) and WAT-only tubes (88±4%). Estimating ${\mathrm{VOL}}_{\mathrm{PDFF}\leq 50\%}$, the BAT-dominant tube demonstrated higher volume of $0.26\;{\mathrm{cm}}^{3}$ than WAT-dominant ($0.16\;{\mathrm{cm}}^{3}$) and WAT-only tubes ($0.01\;{\mathrm{cm}}^{3}$). The presence of BAT exhibits a lower PDFF relative to WAT, thus allowing segmentation of low PDFF tissue for quantification of volume representative of BAT. Future studies will determine the clinical relevance of BAT volume within human depots.

## 1 Introduction

Brown adipose tissue (BAT) generates heat by nonshivering thermogenesis,^1^ facilitates lipid metabolism, and glucose disposal.^2^ These properties have motivated numerous investigators to study the role of BAT^3^^–^^10^ in the development and management of adiposity and its related comorbidities.^11^^–^^21^ F18-fluorodeoxyglucose positron emission tomography computed tomography detects BAT in adults in a thermoactivated state when actively metabolizing glucose^22^ but fails to detect BAT in a thermoneutral state. Recently published data suggest that magnetic resonance (MR), especially multiecho water–fat imaging (WFI), quantifies BAT in its thermoneutral state.^4^^,^^5^^,^^22^^,^^23^^,^^24^^–^^26^ Based on the chemical shift, it reliably discriminates water from adipose tissue and quantifies lipid content. This technique has been applied in clinical and preclinical studies ^24^^–^^26^ by quantifying proton density fat fraction (PDFF) as a potential marker of BAT based on the histologic difference between BAT and white adipose tissue (WAT). The capability of WFI to identify BAT in a thermoneutral state and the lack of ionizing radiation^22^ set the stage for MR to represent the most appropriate platform to longitudinally quantify BAT in children and young adults. BAT is found in very small depots spread out in different body parts, most abundantly in the interscapular and supraclavicular regions mixed with white adipocytes.^4^^,^^5^ Identifying the presence and the amount of BAT in human depots is pivotal and may serve as a target to combat adiposity and its associated diseases. Therefore, the aim of this study is to quantify the average PDFF and the volume of tissue having PDFF ≤50% (${\mathrm{VOL}}_{\mathrm{PDFF}\leq 50\%}$) based on the PDFF distribution of BAT and WAT on the histogram. We hypothesize that BAT-dominant tissue demonstrates lower PDFF value than WAT-dominant tissue and WAT-only because of the higher content of intracellular water and iron in the mitochondria.^5^ For this aim, we performed *ex-vivo* WFI experiments on relatively pure and a mixed preparation of excised interscapular BAT and inguinal/subcutaneous WAT from mice. We present results from *ex-vivo* experiments using two WFI techniques for comparison to address the influence of partial volume on PDFF metrics. The first technique uses low-resolution acquisitions that are corrected for T2* and multipeak lipid spectrum, thus considered “quantitative” (Dixon). The second technique uses high-resolution acquisitions that are not corrected for T2* and multipeak lipid spectrum. The high spatial resolution technique was performed to investigate the influence of partial volume effect on PDFF metrics and to better spatially segment BAT from WAT.

## 2 Material and Methods

All mice procedures were approved by the Committee on Use and Care of Animals and were conducted in compliance with the Institute of Laboratory Animal Research Guide for the Care and Use of Laboratory Animals.

**Fig. 1:**
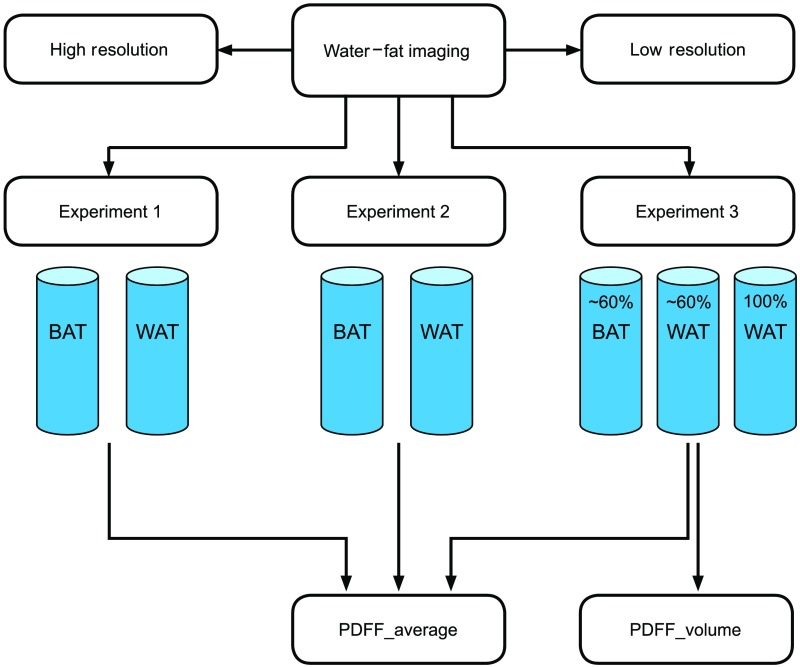
Flowchart of study design. High and low spatial resolution WFI was performed in three experiments. Experiment 1 and 2 used excised murine BAT and WAT packed in individual tubes. Experiment 3 used a mixed preparation of BAT and perigonadal WAT in the first two tubes and subcutaneous WAT in the third tube. The first tube consisted of ∼60% BAT and the second tube consisted of ∼60% perigonadal WAT. PDFF_average was quantified in all three experiments and PDFF_volume was quantified only in the third experiment.

Study design: Preclinical study aimed to quantify the PDFF properties in near homogeneous samples of relatively pure BAT and WAT, as well as inhomogeneous samples containing graded mixtures of BAT with WAT using *ex-vivo* low- and high-resolution WFI experiments (Fig. 1). This experiment allowed us to obtain PDFF from enriched BAT to quantify BAT volume based on the PDFF histogram.

Excised murine BAT and WAT: Adipose tissue was carefully dissected from 20 young adult (8 to 12 weeks old) wild-type male C57bl/6J euthanized mice that were fed a normal diet. The eight euthanized mice that were used in the first *ex-vivo* experiment were 12 weeks old. The eight euthanized mice that were used in the second *ex-vivo* experiment were 8 weeks old and the four euthanized mice used in the third experiment were 12 weeks old. The BAT was harvested from interscapular region in 12 C57Bl/6 male mice and the WAT from gonadal and subcutaneous/inguinal regions in 8 mice. The tissues were placed in a 50 cc conical filled with 1× Dulbecco’s phosphate-buffered saline and transported on ice between laboratories.

Histology: Small portion of BAT and WAT at the time of dissection was used for histological confirmation. Light microscopy and immunohistochemistry (IHC) were performed as described elsewhere.^27^ Uncoupled protein-1 (UCP-1) IHC was performed with antibodies at 10  μg/mL (Clone #536435, R&D Systems MAB6158) to identify BAT. UCP-1 is a marker of metabolic activity of BAT.^3^

*Ex-vivo* experiment setup: Three biological *ex-vivo* imaging experiments were performed. For the first two experiments, the excised interscapular BAT and perigonadal WAT were individually packed tightly in two separate microcentrifuge tubes (35-mm long). For the third experiment, a mixture of excised interscapular BAT and perigonadal WAT was densely packed next to each other in two out of the three microcentrifuge tubes (0.65 cc). The first tube consisted of ∼60% interscapular BAT in the mixture of WAT and BAT. The second tube consisted of ∼60% perigonadal WAT in the mixture of WAT and BAT and the third tube consisted of 100% subcutaneous WAT. We estimated ∼60% composition of tissue by visual inspection while first manually placing the small excised pieces of BAT followed by the pieces of WAT in the vial that was marked at 50%. All tubes were affixed in a 55×75×40  mm box filled with a mixture of water and gadolinium (0.5 mmol ProHance, Bracco Diagnostics, New Jersey) to render strong signal by shortening T1 of water. The samples were set aside to equilibrate to room temperature prior to imaging.

Image acquisition: The *ex-vivo* experiments were performed on a 3-T MR system (Philips Ingenia, Best, The Netherlands) in an 8-channel wrist coil. To investigate the influence of partial volume on PDFF metrics, two standard WFI techniques (low and high spatial resolution) available on the scanner in two orthogonal three-dimensional (3-D) sections (coronal and sagittal) were used for PDFF measurements. Four total acquisitions, such as low and high spatial resolution, in both sagittal and coronal planes were acquired. The first technique uses multiple single gradient-echo per repetition time (TR) high spatial resolution that is not corrected for T2*. The second is 6-gradient-echo quantitative low spatial resolution addressing the T2* bias and multipeak lipid spectrum.^28^ We acquired high spatial resolution WFI to address the influence of partial volume on PDFF metrics and to better spatially segment BAT from WAT. Echo time (TE) was incremented within the high spatial resolution sequence over four sequential acquisitions over 16:54 min (TE=3.8, 4.8, 5.8, and 6.8 ms) for online calculation of water-only, fat-only, and fat-fraction map. This sequence provided higher spatial resolution with the ability to change the acquired voxel size to increase the resolution as opposed to low-resolution quantitative sequence, but the online fat-fraction algorithm did not account for T2* decay. Other technical parameters of the high spatial resolution sequence were: 71 slice 3-D field echo, TR=10  ms, flip=3  deg, NSA=6, FOV=100×60  mm, acquisition $\text{voxel}=0.5\times 0.5\times 2.2\;{\mathrm{mm}}^{3}$ (or $0.5\;{\mathrm{mm}}^{3}$), and reconstructed $\text{voxel}=0.39\times 0.39\times 1.1\;{\mathrm{mm}}^{3}$. The quantitative sequence acquired six echoes within one acquisition over 7:82 min (TE=1.5  ms+n×1.3  ms, n=0,1,…,5) for online calculation of water-only, fat-only, fat-fraction, and T2* maps using a multipeak lipid model. Other technical parameters of the quantitative sequence were: 69 slice 3-D field echo, TR=14  ms, flip=3  deg, NSA=8, FOV=100×60  mm, acquisition $\text{voxel}=1.1\times 1.1\times 2.2\;{\mathrm{mm}}^{3}$ ($2.66\;{\mathrm{mm}}^{3}$), and reconstructed $\text{voxel}=0.39\times 0.39\times 1.1\;{\mathrm{mm}}^{3}$.

Image postprocessing: MRI DICOM data were converted using in-house MATLAB routines for display and segmentation analysis in 3DSlicer. Volumes of interests (VOI) were placed outlining the adipose tissues. Quantitative evaluation was restricted to four central slices, which showed the lowest levels of artifacts (signal void from air around the plastic lead and at the tip of the tubes) for the first two *ex-vivo* imaging experiments and to eight center slices for the third *ex-vivo* imaging experiment. As potential BAT metrics, we quantified the average PDFF and the volume of tissue having PDFF ≤50% (${\mathrm{VOL}}_{\mathrm{PDFF}\leq 50\%}$) based on the PDFF histogram. The PDFF statistics for the first two experiments was recorded as VOI average, standard deviation (SD), minimum, and maximum. The PDFF statistics for the third experiment was averaged over the four acquisitions (two orthogonal 3-D sections in high and low spatial resolution) and recorded as mean±SD. The volume of relative proportion of BAT having PDFF ≤50% over the four acquisitions in the third experiment was estimated using MATLAB based on the PDFF histogram where we observed the separation point between BAT and WAT. We used fat fraction as an estimation of PDFF.

## 3 Results

**Fig. 2:**
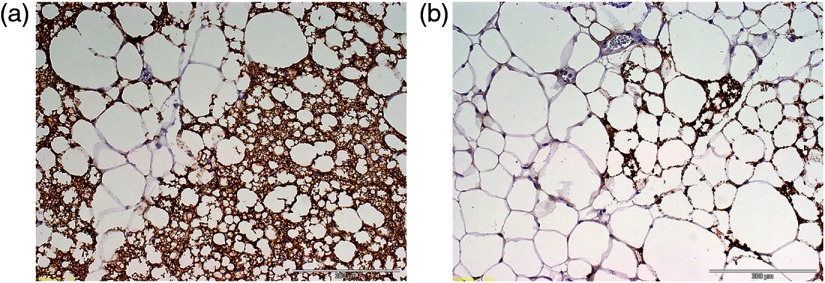
(a) The histology of the BAT demonstrates small brownish adipocytes positive for UCP-1 and (b) the histology of WAT demonstrates large adipocytes negative for UCP-1.

**Fig. 3:**
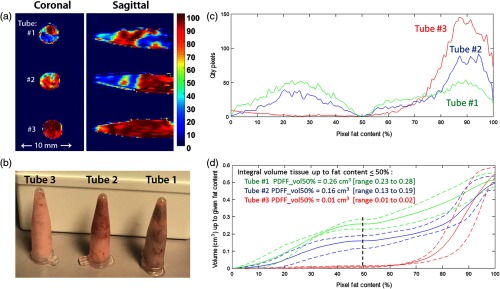
(a) PDFF map and (b) picture of tubes containing mixtures of white and brown adipose: tube 1 is BAT dominant and shows predominantly blue color, tube 2 is WAT (perigonadal) dominant showing predominantly red color, and tube 3 is WAT (subcutaneous) only showing red color. (c) Histograms of PDFF averaged over four distinct acquisition series. (d) Integral volume of histogram up to specified PDFF content of 50% (pixel count×pixel volume) averaged (solid lines), max and min (dash lines) over four distinct acquisition series for tube 1 (green), tube 2 (blue), and tube 3 (red).

Murine BAT and WAT *ex-vivo* imaging experiments: Murine BAT and WAT were confirmed by histology and IHC. The murine BAT consisted of predominantly small adipocytes positive for UCP-1, while the murine WAT consisted of predominately large adipocytes negative for UCP-1 [Figs. 2(a) and 2(b)]. The results of PDFF measurements for the first two *ex-vivo* experiments are listed in Table 1. We found a similar trend of lower PDFF in BAT than in WAT on both quantitative low and high spatial resolution WFI between the first two experiments. The PDFF of WAT was similar between the first two experiments. The second experiment demonstrated excellent reproducibility of the WFI experiment setup. The second experiment showed slightly lower average PDFF of BAT on high spatial resolution image than quantitative low spatial resolution image (21±7.6% and 27±5%) (Table 1). By visual inspection, the BAT corresponded with blue color and the WAT with red–yellow color on color-coded fraction maps [Fig. 3(a)]. The third experiment showed a difference in PDFFs between the tubes containing a mixture of BAT and WAT. The BAT-dominant tube demonstrated average PDFF of 51±35% on high-resolution images that were lower than the WAT-dominant tube with an average PDFF of 74±35% and WAT-only tube with average PDFF of 91±18%. Similarly, the BAT-dominant tube demonstrated lower PDFF of 52±29% on low-resolution images than the WAT-dominant tube with average PDFF of 70±26% and WAT-only tube with average PDFF of 84±12%. The average PDFF of WAT yielded lower values on low-resolution images compared to high-resolution images. To demonstrate robustness of our third experiment, we calculated mean±SD of average PDFFs over the four acquisitions for each tube. The BAT-dominant tube demonstrated lower mean PDFF of 55±2% than WAT-dominant (69±4%) and WAT-only tubes (88±4%). Furthermore, in our third *ex-vivo* experiment, we performed offline histogram analyses to determine the integral volume of relative proportion of BAT within the mixed preparation with WAT up to specified PDFF content over four distinct acquisition series [Figs. 3(a)–3(d)]. Based on the histogram of PDFF averaged over four acquisitions and the BAT distribution within the tissue composition [Fig. 3(c)], we selected a PDFF content up to 50% for segmentation of the BAT. We then estimated the volume of BAT-like tissue by counting pixels having a PDFF value between 0% and 50% (${\mathrm{VOL}}_{\mathrm{PDFF}\leq 50\%}$) within the total volume of $0.6\;{\mathrm{cm}}^{3}$. Tube 1 (BAT dominant) demonstrated higher ${\mathrm{VOL}}_{\mathrm{PDFF}\leq 50\%}$ of $0.26\;{\mathrm{cm}}^{3}$ (range 0.23 to $0.28\;{\mathrm{cm}}^{3}$) compared to tube 2 (WAT dominant) with ${\mathrm{VOL}}_{\mathrm{PDFF}\leq 50\%}$ of $0.16\;{\mathrm{cm}}^{3}$ (range 0.13 to $0.19\;{\mathrm{cm}}^{3}$) and tube 3 (subcutaneous WAT) with ${\mathrm{VOL}}_{\mathrm{PDFF}\leq 50\%}$ of $0.01\;{\mathrm{cm}}^{3}$ (range 0.01 to $0.02\;{\mathrm{cm}}^{3}$) [Figs. 3(a)–3(d)].

**Table 1:** Water–fat MR imaging distinguishes BAT from WAT.

	*Ex-vivo* WFI experiment 1		*Ex-vivo* WFI experiment 2	
PDFF (%)	High resolution	Q Low resolution	High resolution	Q Low resolution
	Mean±SD (min to max)	Mean±SD (min to max)	Mean±SD (min to max)	Mean±SD (min to max)
BAT	25±5 (12 to 37)	23±6 (13 to 45)	21±8 (6 to 39)	27±6 (17 to 40)
WAT	87±7 (66 to 100)	76±7 (65 to 93)	82±5 (61 to 93)	73±6 (56 to 89)

Note: BAT, brown adipose tissue; WAT, white adipose tissue; Q, quantitative; PDFF, proton density fat fraction; and SD, standard deviation.

Note: *Ex-vivo* experiments from euthanized murine BAT and WAT demonstrating differences in PDFF between BAT and WAT. No difference was seen in PDFF between high spatial resolution and quantitative (Q) low spatial resolution multiecho WFI.

## 4 Discussion

Main findings: Our main findings indicate that average PDFF via multiecho WFI can reliably identify the presence of BAT in thermoneutral state and more importantly can quantify the PDFF volume of relative proportion of BAT mixed with WAT. Excellent reproducibility of our WFI experiment setup was seen in the second experiment. In all three *ex-vivo* WFI experiments, the presence of BAT demonstrated lower average PDFF than WAT.

Excised mice adipose tissue *ex-vivo* imaging experiments: Our first two *ex-vivo* WFI experiments of excised murine BAT and WAT demonstrated lower average PDFF of BAT than WAT using both, high- and low-resolution WFI techniques. The lower average PDFF in brown adipocytes comes from the small intracellular lipid droplets, while the higher PDFF in white adipocytes comes from the large intracellular lipid droplet.^5^^,^^10^^,^^23^^,^^24^^–^^26^ It is important to note that *in-vivo*, brown adipocytes are often found contiguous with white adipocytes.^5^^,^^7^^,^^8^^,^^10^^,^^23^^,^^24^^–^^26^ Even though PDFF has been shown to reliably distinguish between BAT and WAT,^23^^,^^24^^–^^26^ in this study, we show the ability of PDFF to quantify the volume of relative proportion of BAT mixed with WAT. Therefore, our third *ex-vivo* WFI experiment of mixture of murine BAT and WAT yields two quantitative variables: average PDFF to determine the presence and of BAT and ${\mathrm{VOL}}_{\mathrm{PDFF}}$ to estimate the integral volume of BAT based on the PDFF histogram up to specified PDFF content. This methodology is sufficient if BAT is being identified in large adipose tissue depots, such as supraclavicular depot, or peri-organ adipose tissue, such as peri-renal or epicardial adipose depot. Therefore, quantifying the relative proportion of BAT and WAT within the depot may potentially become an important clinical marker.^3^^,^^9^^,^^29^ While quantifying PDFF in large adipose depots is feasible, a fundamental limitation of this methodology in small depots is partial volume averaging effects from adjacent organs.^23^^,^^25^ Furthermore, voxels at the interface between adipose tissue and adjacent organs may mimic PDFF that is similar to BAT range.^23^^,^^25^ A priori knowledge of small depots that would suffer from partial volume effect and validation of BAT with histology should mitigate this limitation. This is especially important if the difference in PDFF between two different depots, such as subcutaneous and supraclavicular, is small (82.6% for supraclavicular versus 90.2% for subcutaneous).^9^ In the second experiment, we observed slightly lower average PDFF in high spatial resolution than quantitative low-resolution multiecho WFI. This could potentially be explained by the effect of T2* decay that tends to reduce apparent fat fraction on the high spatial resolution image. Of note, we did not observe the same trend in our first experiment. The age difference of the mice between the two experiments may account for slightly different results. We used slightly older mice (12 weeks of age) in the first experiment than in the second experiment (8 weeks of age). Although 8 to 12 weeks of age is considered young adults for mice, mice that are 12 weeks old may have more white adipocytes in their BAT. However, a similar trend in average PDFF of BAT and WAT was observed between the first two experiments. Our BAT was carefully dissected from the interscapular region of lean mice because the BAT is macroscopically different than the WAT. However, at histology, the BAT contained small amount of white adipocytes. Prakash et al.^5^ have reported slightly increased average PDFF of BAT and slightly decreased average PDFF of WAT in their *in-vivo* preclinical study than our findings of average PDFF of BAT and WAT. *In vivo*, it is often difficult to delineate BAT from WAT, and the partial volume effect from field inhomogeneity may alter PDFF values. Hu et al.^23^ have reported slightly higher but very broad range (40% to 80%) of average PDFF mainly for BAT in mice carcasses and excised tissues using methodology similar to our first two *ex-vivo* imaging experiments. However, this difference is explained by heterogeneous tissue, different scanners, and different preparation techniques.

Prior studies: Hu et al.^23^ and Yokoo et al.^30^ have used a very similar experiment to our first two *ex-vivo* experiments imaging excised murine BAT and WAT by 6-echo IDEAL (General Electric) algorithm that is similar to our low-resolution multiecho water–fat Dixon (Philips) algorithm. The small difference in the average PDFFs between our studies could be contributed to tissue heterogeneity, different scanners, and tissue preparation techniques. Our tissue samples were obtained from the animal laboratory that was five minutes away from the main hospital where imaging experiments were performed. In addition, we performed third *ex-vivo* experiment imaging mixture of BAT and WAT and determined the average PDFF and volume of PDFF of relative proportion of BAT within the mixture. Prior studies have calculated fat mass in muscles,^31^ organs, and adipose tissue^32^ using PDFF approach; however, we quantified the relative proportion of BAT mixed with WAT based on a threshold determined by the PDFF histogram. Our third experiment addresses the challenge of extending this technique for clinical application because in humans, pockets of different amount of BAT are found mixed with WAT and rarely as a single contiguous depot.

Limitations: We performed three *ex-vivo* imaging experiments of euthanized murine BAT and WAT, the first two of which showed excellent reproducibility of this approach, whereas the third addressed partial volume effect of mixtures of tissues. First limitation to translation of this preclinical work is that we imaged excised murine instead of human BAT and WAT. Our third experiment imaging mixture of BAT and WAT brings this concept closer to clinical application because BAT in humans is found mixed with WAT. Second limitation of the translation is the WFI experiment setup. While we cannot translate the same WFI techniques, we will use the same PDFF concept with histogram analysis to quantify the volume of the BAT. The average PDFF of BAT may be lower in live animals than in excised tissue because the difference between room temperature and body temperature influences the fat fraction.^33^^,^^34^ Although we found difference in average PDFF between BAT and WAT, further studies in humans with histologic confirmation of WFI findings should address this limitation.

Future direction: Currently, anatomic imaging is used to quantify the total adipose tissue volume in human depots. This approach does not detect the adipose tissue changes in response to cardiometabolic diseases. Segmenting BAT from WAT volume may represent an important estimate of cardiometabolic health. Future studies should determine the clinical relevance of average PDFF and volume PDFF of BAT as an estimate of obesity phenotypes, risk stratification, and guide management of cardiometabolic disease.

## 5 Conclusion

This work investigates the feasibility of quantifying the volume of relative proportion of BAT mixed with WAT. BAT and WAT show different PDFF behavior. BAT demonstrates lower average PDFF than WAT in relatively pure and in mixed preparation with WAT. It also demonstrates a higher volume of BAT in the BAT-dominant tube compared to WAT-dominant and WAT-only tubes. Multiecho WFI can be used to quantify the presence and the volume of relative proportion of BAT within the depot of interest. These metrics will facilitate longitudinal measurements in human studies; however, future research is needed for validation purposes.
